# Assessing the Utility, Impact, and Adoption Challenges of an Artificial Intelligence–Enabled Prescription Advisory Tool for Type 2 Diabetes Management: Qualitative Study

**DOI:** 10.2196/50939

**Published:** 2024-06-13

**Authors:** Sungwon Yoon, Hendra Goh, Phong Ching Lee, Hong Chang Tan, Ming Ming Teh, Dawn Shao Ting Lim, Ann Kwee, Chandran Suresh, David Carmody, Du Soon Swee, Sarah Ying Tse Tan, Andy Jun-Wei Wong, Charlotte Hui-Min Choo, Zongwen Wee, Yong Mong Bee

**Affiliations:** 1 Health Services and Systems Research Duke-NUS Medical School Singapore Singapore; 2 Centre for Population Health Research and Implementation SingHealth Regional Health System SingHealth Singapore Singapore; 3 Department of Endocrinology Singapore General Hospital Singapore Singapore

**Keywords:** clinical decision support system, artificial intelligence, endocrinology, diabetes management, human factors

## Abstract

**Background:**

The clinical management of type 2 diabetes mellitus (T2DM) presents a significant challenge due to the constantly evolving clinical practice guidelines and growing array of drug classes available. Evidence suggests that artificial intelligence (AI)–enabled clinical decision support systems (CDSSs) have proven to be effective in assisting clinicians with informed decision-making. Despite the merits of AI-driven CDSSs, a significant research gap exists concerning the early-stage implementation and adoption of AI-enabled CDSSs in T2DM management.

**Objective:**

This study aimed to explore the perspectives of clinicians on the use and impact of the AI-enabled Prescription Advisory (APA) tool, developed using a multi-institution diabetes registry and implemented in specialist endocrinology clinics, and the challenges to its adoption and application.

**Methods:**

We conducted focus group discussions using a semistructured interview guide with purposively selected endocrinologists from a tertiary hospital. The focus group discussions were audio-recorded and transcribed verbatim. Data were thematically analyzed.

**Results:**

A total of 13 clinicians participated in 4 focus group discussions. Our findings suggest that the APA tool offered several useful features to assist clinicians in effectively managing T2DM. Specifically, clinicians viewed the AI-generated medication alterations as a good knowledge resource in supporting the clinician’s decision-making on drug modifications at the point of care, particularly for patients with comorbidities. The complication risk prediction was seen as positively impacting patient care by facilitating early doctor-patient communication and initiating prompt clinical responses. However, the interpretability of the risk scores, concerns about overreliance and automation bias, and issues surrounding accountability and liability hindered the adoption of the APA tool in clinical practice.

**Conclusions:**

Although the APA tool holds great potential as a valuable resource for improving patient care, further efforts are required to address clinicians’ concerns and improve the tool’s acceptance and applicability in relevant contexts.

## Introduction

Diabetes mellitus is a chronic condition that affects millions of people worldwide. In Singapore, the prevalence of diabetes is estimated to surpass 400,000, with 1 out of 3 individuals at risk of developing the condition [1]. Uncontrolled diabetes can lead to various complications, such as neuropathy, retinopathy, and nephropathy. Diabetes is primarily associated with cardiovascular diseases, particularly ischemic heart disease and myocardial infarction, which account for most of the mortality cases in patients with diabetes [2,3].

Managing diabetes clinically poses a considerable challenge due to its complex nature. The treatment of diabetes involves achieving specific targets, such as optimal control of glycemia, blood pressure, and lipid levels, primarily relying on laboratory tests [4]. Regular review of test results and subsequent treatment adjustments are important in minimizing the risk of long-term complications and aligning with recommended targets [5]. However, the need to monitor multiple laboratory markers during clinical consultations can impose a cognitive burden. Furthermore, incomplete integration of critical patient data into the electronic medical record (EMR) can lead to errors in disease monitoring, compromising the quality of patient care [6].

Evidence suggests that clinical decision support systems (CDSSs) can assist clinicians in effectively monitoring patient data and making accurate and informed treatment decisions [7-9]. Traditionally, CDSSs have relied on medical expertise and clinical practice guidelines. However, keeping CDSS content and knowledge up-to-date is increasingly challenging due to the evolving nature of clinical practices [10]. The advent of big data and machine learning has enabled the development of artificial intelligence (AI)-powered CDSSs, capable of diagnosing conditions, suggesting evidence-based treatment options and aiding in care planning [11,12]. Research shows that AI-powered CDSSs have improved the quality of diabetes care and patient outcomes [12,13].

Despite the positive impacts of AI-driven CDSSs on health care, fewer studies have examined human factors. In addition, several critical issues surrounding AI-powered clinical tools have been brought to attention, including concerns regarding the transparency of underlying algorithms, accountability, data privacy, and limited trust and applicability [10,14]. Although these studies provide essential insights into implementing AI-based CDSSs, a significant gap exists in research concerning the early-stage implementation of an AI-enabled CDSS specifically for type 2 diabetes mellitus (T2DM). Evaluating a CDSS in the early stages of implementation is of utmost importance to optimize its benefits and mitigate potential drawbacks, as it offers vital information on use, acceptability, and the challenges pertaining to human factors in real-world clinical settings.

To support clinicians in making better treatment decisions in T2DM management, the AI-enabled Prescription Advisory (APA) tool was developed and integrated within the endocrinology specialist clinics at the Diabetes & Metabolism Centre in Singapore General Hospital. To ensure that the tool is capable of scaling up and meeting the needs of its users, it is crucial to assess its appropriateness within the clinical context. Therefore, this study aims to explore the perspectives of clinicians regarding the use and impact of the APA tool while also identifying potential challenges associated with its adoption and application.

## Methods

### Overview

This study adopted qualitative research methodology to assess the usability of the tool. For rigor and transparency, we anchored our study according to the COREQ (Consolidated Criteria for Reporting Qualitative Research) checklist [15].

### Development of an AI-Enabled Diabetes CDSS

The diabetes CDSS, also known as the APA tool, was developed using data gathered from the Singapore Health Services Diabetes Registry that comprised a total of 189,520 patients with diabetes. This data set included 6,407,958 outpatient visits spanning over 5 years from 2013 to 2018 [16]. For model development, 80% of the data set was used to build therapeutic recommendations, while the remaining 20% was used to test and validate the trained models. Three distinct therapeutic recommendation models were formulated for antiglycemic, antihypertensive, and lipid-lowering treatments. These models were created by integrating both a knowledge-driven approach and a data-driven approach. The knowledge-driven approach, initially drawing inputs from clinical guidelines and expert opinions, was used to identify potential therapeutic options. Subsequently, the data-driven approach that used deep learning techniques was used to select the identified therapeutic options based on anticipated clinical outcomes. To assess the performance of model’s prediction, short-term outcomes compared therapeutic options between treatments that aligned with the model’s recommendations and those that did not. Confounding factors were also accounted along the way and adjusted by stratification and multivariate regression. For evaluation of long-term outcomes, the rates of model-concordant treatments were computed by multivariate logistic regression to determine whether the combined treatments exhibited a positive impact on reducing the occurrence of long-term complications and mortality.

### Features of the AI-Enabled Diabetes CDSS

**Figure 1:**
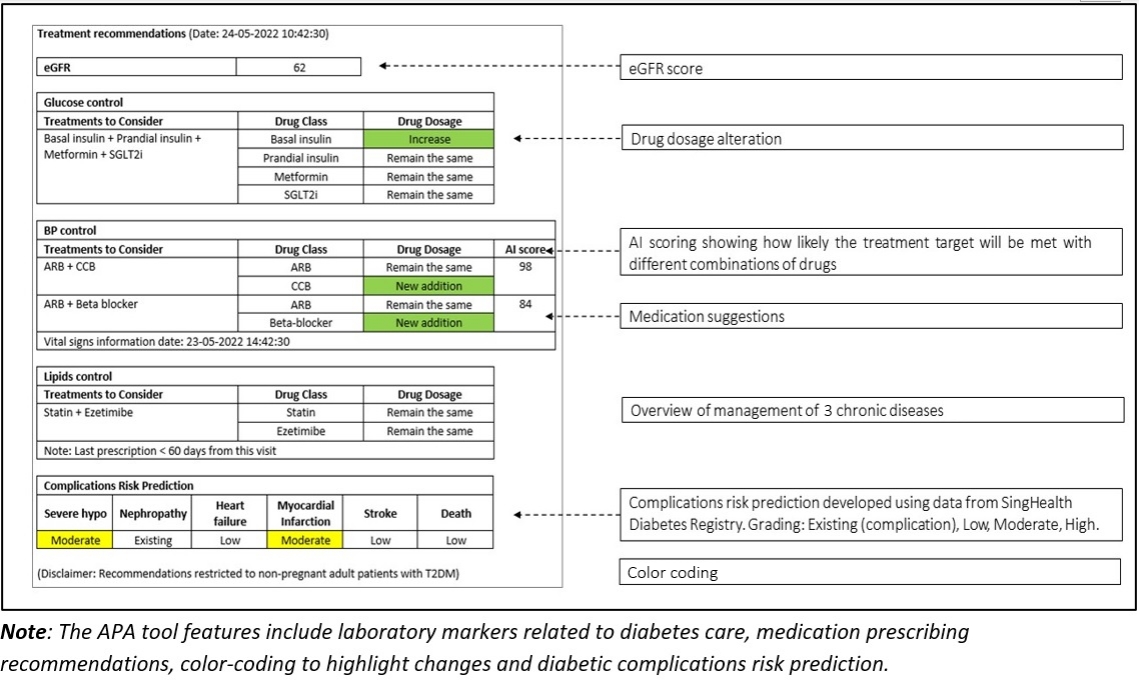
Outputs of APA tool. The APA tool features include laboratory markers related to diabetes care, medication prescribing recommendations, color-coding to highlight changes, and diabetic complications risk prediction. AI: artificial intelligence; APA: AI-enabled Prescription Advisory; ARB: angiotensin receptor blockers; BP: blood pressure; CCB: calcium channel blockers; eGFR: estimated glomerular filtration rate; T2DM: type 2 diabetes mellitus.

Presently, the APA tool has been integrated into the EMR system at the Singapore General Hospital to provide tailored treatment recommendations for achieving target glucose, blood pressure, and cholesterol levels. It features 3 distinct AI components designed to improve patient outcomes and support clinical decision-making. The first component recommends drug classes based on laboratory markers such as glycated hemoglobin (HbA_1c_), low-density lipoprotein cholesterol, and blood pressure measurements, aiding clinicians in selecting the most appropriate drug classes to achieve glycemic, low-density lipoprotein cholesterol and blood pressure treatment targets. The second component generates an AI score that indicates the likelihood of reaching treatment targets when adopting a suggested new drug therapy. The third component generates AI-based diabetic complications risk predictions, providing specific complication risks associated with suboptimal diabetes treatment. All outputs generated by the AI model are color-coded for enhanced visibility. Figure 1 provides a snapshot of the APA tool’s outputs, illustrating how clinical results extracted from a patient’s EMR are presented.

### Participants

Eligible participants were (1) clinicians trained in endocrinology, (2) currently employed full-time by the institution, (3) completed training in APA, and (4) used the APA for a minimum duration of 4 weeks. We purposively selected participants according to age, gender, and seniority level in the workplace to gain a range of perspectives. Participants were approached via email, and informed consent was obtained prior to enrollment into the study.

### Study Procedure

Prospective participants were purposefully selected and approached by the research team to ensure their engagement in the study. Consented participants took part in a comprehensive group training session, encompassing the following key components: (1) a comprehensive overview of the tool’s development and validation process, (2) exploration of the specific features and underlying knowledge rules that inform the therapeutic recommendations, (3) familiarization with the tool’s output (ie, therapeutic recommendations), and (4) an interactive question and answer segment addressing general inquiries. The entirety of the training session was conducted over the course of 1 hour. Following the training, the participants were granted immediate access to the APA tool during their clinical consultations. It was emphasized that the participants had the freedom to decide on the final treatments for their patients with T2DM, regardless of the clinical recommendations provided by the APA tool. Each participant was given a minimum of 4 weeks of exposure to the tool in the clinic setting before his or her involvement in this study. Following the 4-week period, the participants were invited to partake in a focus group discussion (FGD).

### Data Collection

A semistructured FGD guide was developed by drawing upon relevant literature and leveraging the expert knowledge of the study team [17,18]. FGD was chosen to foster a dynamic and interactive environment that encouraged the exploration of shared experiences and perspectives within the professional context, thereby providing a comprehensive understanding of the collective viewpoints users had on the APA tool. To attain a variety of opinions, the participants were recruited according to their seniority (registrars, associate consultants, consultants, and senior consultants). FGDs were conducted exclusively among participants holding equivalent hierarchical positions within the workplace, with the specific intention of minimizing the potential for power differentials and fostering open and candid dialogue. Key topics of interest included (1) participants’ firsthand experiences while using the APA tool, (2) perceptions and evaluations of the various features, (3) impacts of the APA tool on clinical practice, and (4) challenges related to the adoption and application. The interview guide underwent pilot-testing multiple iterations. Consented individuals were then invited to participate in virtual FGD (2-6 participants per session according to seniority) over Zoom (Zoom Video Communications, Inc) by a facilitator (HG) trained in qualitative research methodology. Reflections were recorded after each FGD to capture and document valuable insights shared during the discussions. The duration of the FGD ranged from 50 minutes to 75 minutes.

### Data Analysis

Interviews were audio-recorded following verbal consent and transcribed verbatim. Transcripts were checked for validity, and any identified errors were corrected. Two coders (SY and HG) reviewed the transcripts independently and thematically analyzed the data using NVivo (version 12; Lumivero). We used reflexive thematic analysis following each completed interview [19], contributing to the ongoing refinement and direction of the interview guide for subsequent interviews. The coding categories evolved from initial open coding to more analytical coding of the text, ultimately revealing a series of interconnected themes and patterns. The analysis and interviews continued until no new emerging themes were identified. In case of discrepancies, iterative discussions involving study team members were conducted to resolve any differences and ensure consistency in the analysis process.

Recognizing the inherent influence of the coders’ subjective perspectives in the research process, the study team prioritized strategies aimed at effectively managing these preconceptions and upholding the integrity and credibility of the analysis. Specifically, we implemented the following measures: (1) the establishment of an elaborate coding protocol, meticulously designed to promote consistency and minimize potential subjective interpretations and (2) regular engagement in peer-debriefing sessions and member checking to validate the interpretations and enhance the credibility and confirmability of our findings.

### Ethical Considerations

Ethics approval was obtained from SingHealth Centralized institutional review board (2022/2329). Prior to the FGD, a proficient research coordinator (HG) engaged with each participant individually to meticulously review the participant information sheet and consent form. Particular emphasis was placed on elucidating the study’s objectives, along with an extensive exploration of potential foreseeable risks and benefits. Upon satisfactory comprehension, the participants were invited to provide their informed consent by endorsing the documentation. Furthermore, they were duly informed that all collected data would undergo a stringent deidentification process to preserve anonymity. To uphold transparency and equity in the compensation process, the participants were explicitly notified beforehand that no form of compensation would be provided for their involvement in this study. It was reiterated that participation in the study was entirely voluntary.

## Results

### Characteristics of Participants

In total, 18 clinicians were contacted, and 13 responded positively to the invitation and participated in the FGDs. The remaining 5 clinicians declined to participate, citing time constraints and lack of interest as the reasons. The FGDs were conducted based on participants’ seniority at work. Most participants were male (n=8, 61%) and aged between 31 and 50 years (n=12, 92%). The participants held various designations in their respective roles, including resident (n=3, 23%), associate consultant (n=1, 8%), consultant (n=5, 38%), and senior consultant (n=4, 31%). Notably, more than three-quarters (n=10, 77%) of the participants had no prior experience with AI-enabled CDSSs. Detailed characteristics of the study participants are shown in Table 1.

**Table 1:** Characteristics of participants (N=13).

Characteristics		Participants, n (%)
**Age (years)**		
	31-40	6 (46)
	41-50	6 (46)
	Older than 50 years	1 (8)
**Sex**		
	Female	5 (38)
	Male	8 (61)
**Seniority at work**		
	Resident	3 (23)
	Associate consultant	1 (8)
	Consultant	5 (38)
	Senior consultant	4 (31)
**Prior experience with AI^a^-enabled CDSSs^b^**		
	Yes	3 (23)
	No	10 (77)

^a^AI: artificial intelligence.

^b^CDSS: clinical decision support system.

Our analysis yielded 2 themes and 9 subthemes that represented the participants’ perspectives concerning the use and impact of the APA tool on patient care and clinical practice and the challenges to adoption and application of the APA tool. Descriptions of themes and subthemes are presented in Textbox 1.

**Main themes and subthemes.**

**Use and impact of the AI–enabled Prescription Advisory (APA) tool on patient care and clinical practice**

- Supporting decision-making for patients with comorbidities (artificial intelligence [AI]–powered drug class recommendations)
- Facilitating doctor-patient communication (diabetic complications risk predictions)
- Enhancing clinical confidence through cross-checking (color-coded AI-generated recommendations)
- Serving as a gatekeeper against medical negligence

**Challenges concerning adoption and application of the APA tool**

- Interpretability issues due to the lack of standardized guidelines on AI risk predictions
- Mistrust in the system driven by perceived lack of transparency around system development and information sourcing
- Limited applicability in a specialist setting given extensive expertise and patient care accountability of endocrinologists
- Concerns about potential harm in light of occasional contradictions between the APA tool recommendation and a clinician’s professional judgment
- Frustration with technical issues associated with the tool implementation

### Use and Impact of APA Tool on Patient Care and Clinical Practice

When asked about their experience with the APA tool, most participants expressed a positive impact, highlighting its potential to guide clinical decision-making as a key benefit. Specifically, the participants appreciated the AI algorithm’s ability to provide drug class recommendations based on patients’ laboratory markers. Notably, the tool not only simplified chronic disease management but also assisted in identifying instances of suboptimal disease control that might have otherwise gone unnoticed during consultations. This streamlined approach proved invaluable in guiding clinicians toward effectively managing comorbidities and reducing the risk of long-term complications in patients with diabetes.

I think the most valuable part for me is the lipid control feature. Sometimes when engrossed in discussing patients' diabetes treatment plans, which is anyway their primary reasons for seeking consultation, I may overlook the assessment of their LDL-c levels. With the APA tool, a quick glance provides a clear indication of whether they are on target or not. There isn't much extra clinical information that is required by the tool, so I am able to rely on the medication recommendations to appropriately adjust the medication for hyperlipidemia.

FGD 2, senior consultant

Furthermore, some participants saw the AI-generated complication risk predictions as a helpful resource in “convincing patients to adhere to certain treatments or treatment plans.” By presenting visible evidence regarding the potential risks linked to noncompliance or inadequate treatment, the tool showed considerable potential in facilitating doctor-patient communication based on risk prediction.

The complications risk prediction feature stands out as particularly beneficial to me. For example, it provides an alert regarding the risk of hypoglycemia. When the risk level is classified as moderate or high, this information helped me better persuade patients to consider specific treatments or to improve their compliance with the recommended approach.

FGD 2, senior consultant

Some participants pointed out the lack of quantitative representation for the AI-generated complication risk prediction scores. They proposed an interactive time series graph that would visually illustrate the fluctuations in risk scores over time following the adoption of the tool’s recommendations. Participants believed that integrating visual aids would enhance patients’ understanding of their current risk levels associated with complications and promote the benefits of adhering to treatment plans.

The ability to visually present individual risk in a quantitative way through graphical or pictorial means and illustrate the potential changes that may occur after adopting the systems' recommendations would improve information delivery. I personally believe that patients are more inclined to accept the recommendations when they see their risk in a pictorial or a graphical format.

FGD 1, associate consultant

The color-coded AI-generated recommendations served as an additional point of reference during consultations, particularly when discrepancies emerged between the tool’s recommendations and the clinician’s own knowledge. This feature not only fostered critical thinking but also prompted clinicians to consider additional clinical histories that might have been overlooked initially. Overall, clinicians reported an enhanced level of confidence in their clinical decisions, thereby “improving the quality of patient care.”

So, the tool helps to reinforce my decision-making. The color-coded recommendations provide a clear visual indication, prompting me to address any discrepancies that may arise between the tool's suggestions and my own clinical plan. In this case, I delve into additional clinical histories that the tool does not have access to and elucidate the rationale behind my decisions. This process enhances my confidence and guides better decision-making during the clinical visit, which can improve the quality of patient care.

FGD 1, consultant

By and large, the participants perceived the APA tool as a mechanism to prevent the risk of negligence, especially in fast-paced clinical environments. Acting as a “gatekeeper for patient safety,” the APA tool effectively identified and flagged abnormal results, mitigating the risk of overlooking important tasks. The tool was regarded as a valuable partner in pursuit of delivering high-quality and safe care to patients.

I like the idea of the tool as a gatekeeper for patient safety. Making sure doctors don't forget things, reminding us to check and act on abnormal results. I think that is useful for busy clinics.

FGD 1, associate consultant

### Challenges to the Adoption and Application of APA Tool

Although participants generally acknowledged the beneficial effects of the APA tool on quality patient care and clinical practice, they equally expressed reservations about incorporating and using the tool in their own clinical settings. One major concern centered around the interpretability of the automatically generated AI score when new drugs were recommended. While participants appreciated the availability of the scoring system to inform the likelihood of achieving treatment targets based on the recommendations, they remained unsure about the interpretability of the AI score.

I think it is quite interesting that the system is able to provide different percentages of achieving optimum blood pressure when different combinations of new drugs are used. However, my question is if plan A gives a score of 48 while plan B gives a score of 45, are these recommendations still clinically relevant? I mean, of course, the situation is more direct in cases with scores such as 98 and 88, then it will make more sense to pick the plan with 98% of likelihood.

FGD 1, consultant

A sense of mistrust in the APA system emerged, which appeared to stem from the unfamiliarity surrounding AI-based recommendations and concerns regarding transparency of the information sourcing and system development. Some participants openly expressed their hesitancy in adopting the APA tool due to the absence of essential clinical data. Without access to this information, they were not confident enough to use the tool.

When it [APA] was launched, a lot of us were not very sure how it was developed. I think part of the reason why we did not use it very much is also because we are not so familiar with how this system came about, what kind of information was used, and where the information came from. Is it also possible that critical information was not captured in the system? I can't trust totally, and [I am] not confident with what I'm seeing at the moment.

FGD 3, senior consultant

However, participants expressed openness to embrace the tool if they were presented with additional information. They emphasized the importance of transparent communication regarding the evidence supporting the system and the sources of information used. By gaining a clearer understanding of the logic and rules behind the recommendations, they would be more inclined to use the tool in their clinical practice.

[T]hat being said, if more information or transparent communication is given to us, I might be more inclined to use it in clinics. As I know the logic and rules behind these recommendations and where they are sourced from.

FGD 3, senior consultant

While a minority, some clinicians exhibited strong confidence in their own clinical judgment and thus did not see the necessity to rely on the APA tool. They felt that their experience and specialist training surpassed the assistance provided by the AI-driven system. In addition, they highlighted the potential ramifications of relying on CDSS recommendations, emphasizing that the responsibility for patient outcomes ultimately rested with the clinician. Consequently, this attitude led to a reluctance to use the tool, particularly among those who believed that they possessed the requisite expertise to make well-informed decisions in patient care.

I would say that I'm as good or even better than the system. I don't feel the need to rely on it; I'll just do what I do. We are all trained endocrinologists, so we trust our judgment because that has been our bread and butter for many years. At the end of the day, we bear the responsibility for our patients, so you know, if the algorithm makes a sound decision, but something unfortunate ever happens to the patient, then it's still our own accountability on the line.

FGD 1, consultant

These clinicians suggested the potential for the APA tool to bring benefits to the wider primary care community, particularly those who may be “less familiar with endocrinology clinical practices.” They believed that the tool could assist general practitioners in effectively managing patients with complex cases and improve patient engagement.

These recommendations would be more valuable in a primary healthcare setting, where doctors may not have extensive knowledge of clinical practices related to novel glucose-lowering medications and insulin titration, especially in complex cases. I think implementing the AP tool in such settings would greatly help doctors in improving patient engagement and care.

FGD 1, consultant

Another important theme was related to the potential harm of the APA tool’s drug recommendations on patients. Participants noted that the recommendation occasionally contradicted their own professional judgment. They cautioned against solely relying on algorithmic recommendations for clinical decision-making.

Some of the recommendations go against your clinical judgement. For example, I have two patients and the AI recommendation was to add a beta blocker to someone who doesn't have ischemic heart disease as a second line agent. That's just not something that we would normally do. So have to exercise caution too!

FGD 1, consultant

Finally, the participants expressed their frustration with the technical issues associated with the integration of the APA tool into the EMR system. The slow loading of clinical notes resulted in delayed clinical consultations, which added unnecessary mental burdens for some participants. Moreover, there were instances in which the clinical notes failed to load entirely, thereby affecting the quality of patient care.

One significant issue we encounter after implementing the CDSS is the considerable lag in loading clinical notes. It takes a few minutes to retrieve the clinical notes. So, by that time, I'm typically already engaged in a conversation with the patient, and we may even come up with a plan without the notes being available. In some instances, I can't even see the clinical notes at all.

FDG 4, registrar

## Discussion

### Principal Findings

This qualitative study explored clinicians’ perspectives on the use and impact of the APA tool, as well as challenges to its adoption and application in clinical practice. In terms of use, the APA tool offers several useful features to assist clinicians in effectively managing diabetes. As shown in the literature, patients with T2DM frequently experience multiple comorbidities, which may add complexity to pharmacotherapy management and increase the mental burden of prescribing practices [20]. Our findings suggest that the AI algorithms for drug alteration embedded in the APA tool were generally viewed as a good knowledge resource in supporting the clinician’s decision-making on drug modifications at the point of care, particularly for patients with T2DM with comorbidities.

Complications arising from diabetes pose a significant burden on the public health care system [21,22]. In light of this, an important feature developed in the APA tool was the diabetic complications risk prediction that provides information on the likelihood of developing the 6 most common diabetic complications in patients with T2DM [23,24]. We found that participants viewed the risk prediction as having a positive impact on patient care by facilitating early doctor-patient communication and initiating prompt clinical responses to delay the progression of complications associated with diabetes. This finding is similar to that of other research that AI-enabled CDSSs had a positive impact on patient-provider encounters and shared decision-making [25,26]. Therefore, appropriate use of risk prediction could enable clinicians to take early proactive measures to reduce the risk of developing diabetic complications, ultimately reducing the health care costs associated with diabetes [27,28].

Despite the perceived merits of AI-generated risk scores, the absence of clear frameworks (or the scientific basis from which recommendations were derived) limited the interpretability and usability of the risk scores and subsequent follow-up actions. This has been similarly identified in the literature as a key hindrance to clinical adoption [29,30]. As knowledge is deciphered differently based on personal experience and beliefs, the interpretation of scores could be dependent on the subjective attitudes of clinicians in decision-making [31]. Indeed, recent research indicates that the varying levels of knowledge and self-reported behavior among clinicians affect their approach in clinical practice, leading to potential noncompliance with the system recommendations [13]. Furthermore, as shown in our study, some clinicians chose to abstain from using the APA tool entirely because of their lack of trust in the quality of model inputs and parameters, as well as their concerns regarding the logic behind the AI outputs, often referred to as the “black box” situation [32,33]. To ensure a successful expansion of the APA tool within the clinical ecosystem, more effort should be directed to obtain a better comprehension of clinicians regarding the AI technology’s capabilities and the use of explainable frameworks to enhance transparency and clinician engagement [34-36].

As with the literature, clinicians in our study cautioned against being overly reliant on the APA tool, as occasional erroneous recommendations generated by the systems might prompt users to override a correct decision they have already made [26,37]. When users are subjected to automation bias, a tendency to overaccept system recommendations as a heuristic replacement of vigilant information processing [38] and medical errors ensue from following incorrect recommendations. Not only does it predispose patients to even greater harm, but it also diminishes the intention of using AI-enabled CDSSs [39]. Our results underscore the importance of collaborative intelligence, where users and AI work synergistically to enhance patient care. The human-in-the-loop concept suggests that while human oversight is active, overdependence on AI-enabled CDSSs is equally harmful. The optimal approach involves granting clinicians full control over the decision-making process while using AI to offer recommendations and inputs [40]. Clinical decisions, therefore, cannot be made without active involvement from clinicians to serve as gatekeepers, prevent negligence, and ensure patient safety. The seemingly conflicting recommendations identified in this study should be viewed as a catalyst that prompts critical thinking, and more effort should be made to confront meaningful disagreements. Also, encouraging clinicians to check on discrepancies may enhance their confidence in decision-making [41].

Finally, a significant obstacle that hindered the adoption of APA tool pertains to concerns surrounding accountability and liability, which is in line with the literature [36,42]. While ethical considerations regarding the use of AI persist, establishing well-defined clinical standards and codes of conduct for adopting APA tools can foster a culture of shared responsibility, moving away from a single form of attribution of responsibility. In addition, instead of focusing on assigning blame, it would be more constructive to acknowledge and commend clinicians’ efforts in integrating the outputs of AI-enabled CDSSs into their decision-making process, as long as the adoption of recommendations adheres to clinical standards, legal obligations, and ethical principles. This approach would motivate clinicians to embrace the most advanced medical technologies available in clinical practice, even if it means having to make a judgment call.

Collectively, the findings underscore the promising impact of adopting the APA tool within clinical settings and its potential to usher in notable enhancements in health policy. While the rapid integration of AI-based CDSSs in health care has presented promising potential for improved patient outcomes and streamlined clinical workflows, the persistent liability concerns among clinicians have created a barrier to the widespread adoption of these advanced technologies. With clinicians ultimately bearing the responsibility for any medical negligence, even after consulting with AI-enabled CDSS recommendations, there arises an urgent need for a comprehensive medicolegal framework. Such a framework must emphasize the allocation of liability among users, while also ensuring transparency in the decision-making processes of these AI tools [43]. For instance, the policy should delineate clear protocols for the documentation of AI-based recommendations, ensuring that the decision-making process is well-documented and easily accessible for medicolegal reviews. In addition, it is crucial to establish standardized protocols for the continuous evaluation and improvement of AI algorithms to minimize the risk of errors and improve the accuracy of recommendations. Creating an environment that fosters trust in AI technologies through a robust medicolegal framework will ultimately encourage clinicians to embrace these tools, leading to enhanced health care delivery and patient outcomes.

### Limitations

This study provides valuable insights into the benefits and adoption challenges of an AI-based CDSS in its early stage of implementation. This study has some limitations. The study participants were limited to endocrinologists in a tertiary hospital; therefore, the generalizability of the findings to other health care settings may be limited. The sample size of the study is small, which may hinder the generation of comprehensive insights that better represent the broader context. As adoption of AI technology in clinical settings is still in its early stage, assembling a large cohort of clinicians for an in-depth analysis of AI-enabled CDSS implementation can be challenging due to the limited number of early adopters. Nevertheless, our findings shed light on the initial experiences and perceptions of a key group of clinicians, offering a foundation for future research and more extensive investigations. Our study’s sample size also aligns with the systematic review, which found that empirical studies, especially those with homogenous populations and narrowly defined objectives, typically achieve data saturation with 9-17 interviews [44]. Further research is needed to explore the use and impact of the APA tool in different clinical settings, such as primary care. As suggested by our participants, the use of the APA tool can be particularly beneficial to general practitioners who are responsible for managing a wide range of conditions and require access to a breadth of knowledge base across various specialty areas. Despite early findings on the APA tool’s use and adoption challenges, its long-term impacts on clinical and economic outcomes remain unknown. A subsequent larger evaluation is warranted to compare the APA tool with a standard of care. Finally, we did not explore the perspectives of patients with diabetes as the important end users of the APA tool. Incorporating their perspectives may have contributed to a richer understanding.

### Conclusions

AI-enabled CDSSs, such as the APA tool, has the potential to enhance clinical practice and patient care. Clinicians found certain features such as AI algorithms on medication adjustment and complication risk predictions useful in managing patients with T2DM with comorbidities and facilitating doctor-patient communication. However, interpretability of the risk scores, concerns about overreliance and automation bias, and issues surrounding accountability and liability were commonly cited as challenges inhibiting the adoption and application of the APA tool in endocrinology clinical settings. Further work is required to address these concerns effectively to enhance the tool’s acceptance and applicability in relevant contexts.

## Abbreviations

AI: artificial intelligence
APA: AI-enabled Prescription Advisory
CDSS: clinical decision support system
COREQ: Consolidated Criteria for Reporting Qualitative Research
EMR: electronic medical record
FGD: focus group discussion
T2DM: type 2 diabetes mellitus

## Data Availability

The data that support the findings of this study are available upon reasonable request from the corresponding author. The data are not publicly available due to information that could compromise the privacy of research participants.
